# Cognitive Neuroscience and Causal Inference: Implications for Psychiatry

**DOI:** 10.3389/fpsyt.2016.00129

**Published:** 2016-07-19

**Authors:** Nadine Dijkstra, Leon de Bruin

**Affiliations:** ^1^Donders Institute for Brain, Cognition and Behavior, Radboud University Nijmegen, Nijmegen, Netherlands; ^2^Department of Philosophy, VU University Amsterdam, Amsterdam, Netherlands

**Keywords:** interventionism, causal exclusion problem, cognitive neuroscience, psychiatry, mental causation

## Abstract

In this paper, we investigate to what extent it is justified to draw conclusions about causal relations between brain states and mental states from cognitive neuroscience studies. We first explain the views of two prominent proponents of the interventionist account of causation: Woodward and Baumgartner. We then discuss the implications of their views in the context of traditional cognitive neuroscience studies in which the effect of changes in mental state on changes in brain states is investigated. After this, we turn to brain stimulation studies in which brain states are manipulated to investigate the effects on mental states. We argue that, depending on whether one sides with Woodward or Baumgartner, it is possible to draw causal conclusions from both types of studies (Woodward) or from brain stimulation studies only (Baumgartner). We show what happens to these conclusions if we adopt different views of the relation between mental states and brain states. Finally, we discuss the implications of our findings for psychiatry and the treatment of psychiatric disorders.

## Introduction

Traditionally, cognitive neuroscientists have been probing the relation between brain states and mental states by manipulating the mental state of the participant through different conditions and then measuring the associated changes in neural activity, for example by means of Functional Magnetic Resonance Imaging (fMRI) or Electro-encephalogram (EEG). The results of these manipulations are usually taken to reflect a *correlation* between mental states and brain states, rather than a “genuine” *causal* relation. According to several neuroscientists, however, new brain stimulation techniques, such as Deep Brain Stimulation (DBS) and Transcranial Magnetic Stimulation (TMS), allow us to go beyond correlations and establish causal relations between mental states and brain states [for a review, see Ref. (1)]. This has important implications for other disciplines in which these techniques become increasingly popular. For example, in psychiatry, DBS has proven to be an effective treatment for patients with major depressive disorder (MDD) who do not respond to pharmacotherapy or psychotherapy (2–4).

In the current paper, we investigate whether and to what extent it is indeed justified to draw conclusions about causal relations between brain and mental states on the basis of cognitive neuroscience studies. In the next section, we start with a description of an interventionist account of causation, which is inspired by Woodward (5). We argue that this account is more or less in line with how causation is understood in scientific practice. The question is, however, whether it can be used to make causal claims about the interaction between mental states and brain states. In order to address this question, we introduce the notion of supervenience in Section “Mental States and Brain States: A Supervenience Relation.” This notion aims to capture the intuition that mental states are dependent on, but not identical with, brain states. In Section “Causation in Traditional Cognitive Neuroscience Studies,” we turn to Baumgartner’s “causal exclusion” argument. According to this argument, the assumption of a supervenience relation violates the criteria of what counts as a good intervention. As a result, we cannot draw conclusions about the causal relation between mental states and brain states. In his reply to Baumgartner, Woodward (6) proposes to adjust these intervention criteria in order to make room for supervenience relations and to secure causal claims on the basis of traditional cognitive neuroscience studies. In Section “Causation in Brain Stimulation Studies,” we discuss the consequences of both positions for causal claims on the basis of brain stimulation studies. Most importantly, we will show that Baumgartner’s causal exclusion argument does not apply to these studies. That is, we can make causal claims about brain stimulation studies *even* if we assume a supervenience relation and accept Woodward’s original intervention criteria. In Section “Articulating the Mind–Brain Relation,” we show what happens to these conclusions if we adopt a different view of the relation between mental states and brain states. Finally, in Section “Conclusion,” we briefly discuss the implications of our findings for psychiatry and the treatment of psychiatric disorders.

## The Interventionist Account of Causation

In most textbooks on experimental research two main requirements are described that an experiment must meet to be able to reveal a causal relation between *X* and *Y*. The first is that the levels of *X* must be systematically varied and the second is that all variables other than *X* and *Y* are to be controlled in order to eliminate other possible causes of *Y*. If these requirements are met and changes in *X* are accompanied by changes in *Y*, one is allowed to speak of a causal relation between *X* and *Y* (7, 8).

This notion of how to investigate causal relations in scientific practice is very much in line with a philosophical account of causation that has become quite popular recently: interventionism. One of the most established interventionist definitions of causation comes from Woodward (5):

(M) A necessary and sufficient condition for *X* to be a (type-level) *direct cause* of *Y* with respect to a variable set **V** is that there be a possible intervention on *X* that will change *Y* or the probability of *Y* when one holds fixed at some value all other variables *Z_i_* in **V**. A necessary and sufficient condition for *X* to be a (type-level) *contributing cause* of *Y* with respect to variable set **V** is that (i) there be a directed path from *X* to *Y* such that each link in this path is a direct causal relationship… and that (ii) there be some intervention on *X* that will change *Y* when all other variables in **V** that are not on this path are held fixed [Ref. (5), pp. 59].

We mainly focus on the definition of a direct cause since this comes closest to the notion of causation as it is investigated in scientific practice (i.e., it explicitly involves the two requirements mentioned above). However, for the definition to make sense, we also need a clear notion of what an appropriate intervention is. Woodward (5) defines an intervention variable as follows:

(IV) *I* is an intervention variable for *X* with respect to *Y* if:

1. *I* causes *X*;
2. *I* acts as a switch for all the other variables that cause *X*. That is, certain values of *I* are such that when *I* attains those values, *X* ceases to depend on the values of other variables that cause *X* and instead depends only on the value taken by *I*;
3. Any directed path from *I* to *Y* goes through *X*. That is, *I* does not directly cause *Y* and is not a cause of any causes of *Y* that are distinct from *X* except, of course, for those causes of *Y*, if any, that are built into the *I–X–Y* connection itself; that is, except for (a) any causes of *Y* that are effects of *X* (i.e., variables that are causally between *X* and *Y*) and (b) any causes of *Y* that are between *I* and *X* and have no effect on *Y* independently of *X*.
4. *I* is (statistically) independent of any variable *Z* that causes *Y* and that is on a directed path that does not go through *X* [(5), pp. 98].

Finally, relative to the notion of an intervention variable an (actual) *intervention* can be straightforwardly understood in terms of an intervention variable *I* for *X* with respect to *Y* taking on some value *z_i_* such that *I* = *z_i_* causes *X* to take on some determinate value *z_j_* [(5), pp. 98]. In terms of experimental design, an intervention can be seen as a manipulation that changes the variable *X*. In order for this manipulation to be able to reveal a causal relation, it has to meet the requirements in (IV).

## Mental States and Brain States: A Supervenience Relation

Can we use interventionism to make causal claims about the interaction between mental states and brain states? To answer this question, we will (initially) assume a very minimal relation between mental states and brain states – one that captures the intuition that mental states are dependent on brain states. In the philosophy of mind, this relation is known as “supervenience.”

**Figure 1:**
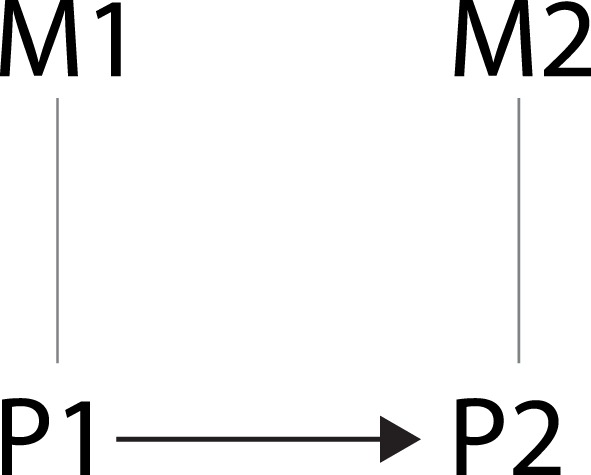
**Schematic representation of the relation between brain states and mental states. Undirected edges indicate supervenience relations and the arrow indicates a causal relation**.

A schematic representation of a supervenience relation between mental states M1 and M2 and brain states P1 and P2 is depicted in Figure 1. Although the notion of supervenience has been much discussed, there are two features that are common in most definitions:

(S1) ¬(*M* causes *P*) ∧ ¬(*P* causes *M*);
(S2) Every change in the value of M is necessarily accompanied by a change in the value of P.

This means that (i) supervenience is a non-causal relation such that neither M causes P nor vice versa^1^ and (ii) any change in mental state is necessarily accompanied by a change in brain state. Furthermore, with regard to Figure 1, we will assume that:

(S3) P1 causes P2.

The end result is a schematic representation of two types of relations: one between *properties* (M1 and P1, M2 and P2), which is captured by a supervenience relation, and one between *events* (M1/P1 and M2/P2), which is captured by a causal relation (i.e., event 1 causes event 2).

## Causation in Traditional Cognitive Neuroscience Studies

With the interventionist account of causation and the notion of supervenience in place, let us now take a closer look at traditional (non-invasive) cognitive science studies.

**Figure 2:**
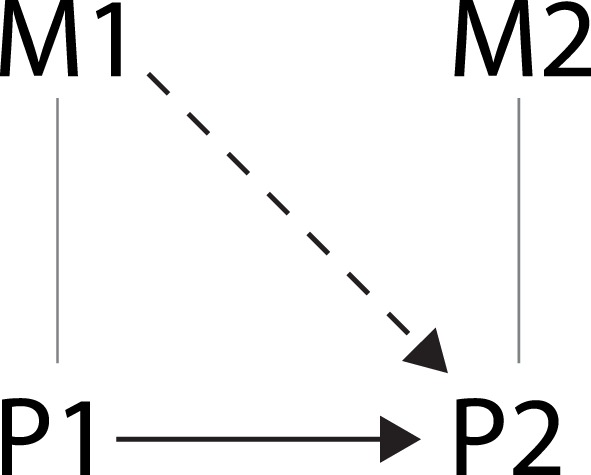
**Schematic representation of the relation investigated in traditional cognitive neuroscience studies indicated by the dashed arrow**.

In most of these studies, the relation between mental states and brain states is investigated by observing the effect of changes in mental state M1 on brain state P2 (see Figure 2). This is done by manipulating the mental state of the subjects by letting them participate in separate conditions that differ on some stimulus characteristic or task that is meant to induce changes in M1. To investigate the effect of these manipulations on brain states, the subjects’ brain activity P2 is measured in all conditions. Then, if the researcher has made sure that the conditions only differ on the manipulated mental variable (using all kinds of controls like randomization of subjects), and a (significant) difference in brain activity between the conditions is found, the researcher concludes that the manipulated mental variable M1 has had an effect on the measured brain state P2.

However, is it valid to conclude that the change in mental state M1 *caused* the change in brain state P2? According to the causal exclusion argument put forward by Baumgartner (9), it is not.

### Baumgartner’s Causal Exclusion Argument

In his argument, Baumgartner (9) takes together the interventionist definition of causation as described above in (M) and (IV) and the supervenience relation as described in (S1–2) to formulate the following conditional:

(BM) If *M1* is causally relevant to *P2* with respect to the variable set **V** = {*M1, M2, P1, P2*}, then there possibly exists a variable *I*_1_ that causes a change in the value (or the probability distribution) of *M1* and is statistically independent of any variable *Z* that causes *P2* and that is on a directed path that does not go through *M1* [(9), pp. 170].

Now we can see that no such variable *I*_1_ can exist. Because of the supervenience relation between *M1* and *P1*, any variable *I*_1_ that causes a change in *M1* also causes a change in *P1* (S2) and this variable *P1* is on a causal path to *P*2 that does not go through *M1* (S3). In other words, every time we perform an intervention on a subjects’ mental state, by manipulating some variable in separate experimental conditions, we also intervene on their brain state. This is not because the change in mental state causes the change in brain state (recall that a supervenience relation is not a causal relation; S1), but because the intervention changes both the mental state and the brain state (S2). In other words, we cannot control the effect of *P1* on *P2*. It follows that we cannot draw any conclusions about the causal effect of the intervention on the mental state. Furthermore, because the relation between *M1* and *P1* is not a causal relation, we also cannot say that *M1* is a contributing cause to *P2*. In the context of an experiment, we would say that *P1* is a confounding variable for which we cannot control, prohibiting any statement to be made about the causal effect of the independent variable on the dependent variable.

### Woodward’s Response

In reply to Baumgartner’s argument, Woodward (6) proposes that when assessing causation in a variable set that includes supervenience relations between variables, it is not necessary to control for or hold fixed the supervenience base. Thus, it is not necessary to control for *P1* when assessing the relation between *M1* and *P2*. According to Woodward, this is because the interventionist account of causation as defined by (M) and (IV) is intended to apply to systems of causal relations in which no non-causal relations (such as supervenience relations) exist. It is not at all clear whether it is applicable to a system in which non-causal relations are present.

**Figure 3:**
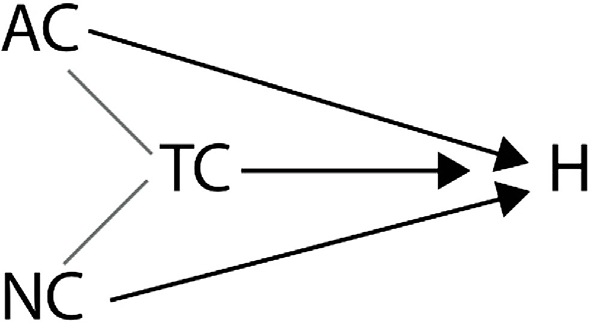
**Schematic representation of the relation between AC, WC, TC, and H**.

Woodward illustrates this by giving an example of a variable set in which non-causal relations are present that are not supervenience relations (6). His example goes along the following lines. Suppose that getting a headache (*H*) is causally influenced by the amount of alcohol consumption (*AC*), which increases the probability of getting a headache, and the amount of non-alcoholic liquid consumption (*NC*), which decreases the probability of getting a headache. We also have a variable representing the total liquid consumption (*TC*), which is the sum of *AC* and *NC*. Assume that we also think of *TC* as causally influencing *H*. We can put all these variables together to get the schematic representation in Figure 3.

**Figure 4:**
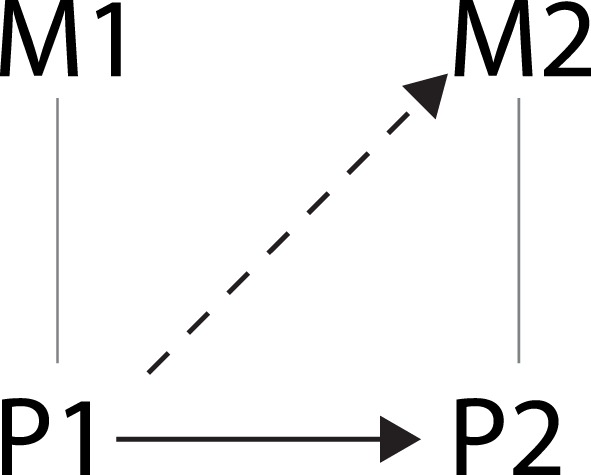
**Schematic representation of the relation investigated in brain stimulation studies as indicated by the dashed arrow**.

Suppose now that we want to investigate if *AC* is causally relevant for *H*. According to Baumgartner’s reading of (IV) and (M), this would mean that it has to be possible to change (intervene on) *AC* without changing any other variable in Figure 4 that is on a directed path to *H* that does not go through *AC*. We can see that this is not possible because *TC* is defined such that if *AC* changes, *TC* also changes. It seems strange to take this finding as evidence for there not being a causal relation between *AC* and *H*. Therefore, Woodward (6) concludes, the interventionist definition as put forward in (M) and (IV) is intended to only apply to systems of causal relations in which no non-causal relations exist. In systems with non-causal relations, one needs to hold fixed only the *appropriate* variables. In the variable set described in Figure 4, this means that if one wants to investigate the effect of *AC* on *H, NC* needs to be fixed, but *TC* does not.

Similarly, Woodward (6) argues, when one wants to investigate the causal effects of supervening variables, their supervenience base does not have to be fixed. This means that *M1* can be causally relevant for *P2* or in other words, according to this interpretation of interventionist causation, investigating the relation between mental states and brain states, as done in traditional cognitive neuroscience studies, by manipulating *M1* and investigating its effect on *P2* can reveal a causal relation between *M1* and *P2*.

In conclusion, according to Baumgartner (9), one cannot draw any conclusions about causal relations between mental states and brain states from traditional cognitive neuroscience studies. However, according to Woodward (6), this is perfectly valid. In the next section, we will discuss both these positions in light of brain stimulation studies in which the brain states are manipulated to investigate the effects on mental states.

## Causation in Brain Stimulation Studies

Since the introduction of brain stimulation techniques such as TMS and DBS, it has become possible for scientists to directly manipulate (intervene on) the electrical activity in the brain. Many neuroscientists have been using these techniques to draw conclusions about the causal relations between brain states and mental states. The following are quotes from TMS and DBS studies published in high-impact journals:

“Making the causal link: frontal cortex activity and repetition priming” (10).

“Causal implication by rhythmic TMS of alpha frequency in feature-based vs. global attention” (11).

“DBS of the subthalamic nucleus markedly improves the motor symptom’s of Parkinson’s disease, but causes cognitive side effects such as impulsivity” (12).

“Stimulation of a restricted site in the upper midbrain can cause major acute depression” (13).

Are these claims justified? In the present section, we will explore this question in light of Baumgartner’s and Woodward’s arguments. Before we continue, we should mention that a large part of the brain stimulation studies only focuses on the effects of changes in brain states on other brain states [e.g., Ref. (14–16)]. This is the relation between *P1* and *P2*. As described in (S3), we assume that there exists a causal relation between these variables. Therefore, in these types of brain stimulation studies, it is perfectly justified to talk about causal effects.

In the brain stimulation studies in which the relation between brain states and mental states is investigated, this seems more complicated. In these studies, the relation between *P1* and *M2* as depicted in Figure 4 is investigated. *P1* is manipulated by stimulating a certain brain area using TMS or DBS in one condition and not stimulating it in another condition, while measuring some mental variable *M2* in both conditions. The researcher tries to make sure that the two conditions only differ on *P* and not on other variables, for example by applying sham stimulation in the control condition. If then a (significant) difference in *M2* between the two conditions is found, the researcher concludes that there was an effect of the change in brain activity on the mental state. However, is he or she justified in saying that *P1* has *caused M2*?

### Baumgartner’s Approach

To determine whether Baumgartner’s argument applies to this experimental set-up, the conditional (BM) has to be redefined. If we switch the relevant terms, we get the following definition:

(BP) If *P1* is causally relevant to *M2* with respect to the variable set **V** = {*M1, M2, P1, P2*}, then there possibly exists a variable *I*_1_ that causes a change in the value (or the probability distribution) of *P1* and is statistically independent of any variable *Z* that causes *M2* and that is on a directed path that does not go through *P1*.

Interestingly, the causal exclusion argument used by Baumgartner (9) to conclude that *M1* is not causally relevant to *P2* does not work in this case. This is because there is no variable in **V** that causes *M2* but does not go through *P1*. It is true that, because of the supervenience relation (S2), any intervention on *P1* also changes *M1*. However, there is no causal relation between *M1* and *M2* that does not go through *P1*. Furthermore, an intervention on *P1* also changes *P2*, through the causal relation mentioned in (S3), but according to (S1), *P*2 does not cause *M2*. So it seems that an intervention on *P1* is possible without intervening on another variable that causes *M*2. This suggests that we actually can make causal claims on the basis of brain stimulation studies, even if we assume a supervenience relation and accept Woodward’s original intervention criteria. Let us now see whether Woodward’s approach leads to a similar conclusion.

### Woodward’s Approach

According to Woodward, if one wants to investigate whether *P1* is causally relevant to *M2*, one needs to perform an intervention to change the value of *P*, while holding fixed all *appropriate* other variables. When investigating the relation between *P1* and *M2*, the supervenience base *P2* is not one of these appropriate variables, so even if *P2* were on a directed path to *M2* that does not include *P1, P2* does not have to be fixed because it is the supervenience base of *M*2.

The other possible candidate for a variable that is on a directed path to *M2* that does not include *P1*, is *M1*. Now this seems to pose a problem. According to Woodward’s adjusted interpretation of interventionist causation, we can argue that *M1* causes *M2*, because *P1* and *P2* do not have to be fixed. This seems to imply that *M1* is an alternative cause for *M2* making it impossible to conclude that *P1* has caused *M2*. However, it seems that in his adaptation, Woodward (6) also argues that supervening variables do not have to stay fixed:

(IV*) An intervention *I* on *X* with respect to *Y* will (a) fix the value of *SB(X)* in a way that respects the supervenience relationship between *X* and *SB(X)*, and (b) the requirements in the definition (IV) are understood as applying only to those variables that are causally related to *X* and *Y* or are correlated with them *but not to those variables that are related to X and Y as a result of supervenience relations* [(6), pp. 32].

This means that *M1* does not have to be fixed in order to draw a causal conclusion about the relation between *P1* and *M2* by intervening on *P1*.

Thus, according to Woodward’s adjusted interpretation of interventionist causation, brain stimulation studies in which appropriate controls are applied, such as randomization of groups and application of sham stimulation in the control group, are suitable to base conclusions about the causal effect of brain states on mental states on.

In conclusion, if one follows Woodward, we can make claims about causal relations between brain states and mental states from the results of both traditional cognitive neuroscience and brain stimulation studies. However, for this to work, we do have to adjust the original interventionist criteria (5) and accept a non-causal supervenience relation. According to Baumgartner, by contrast, we cannot make claims about causation from the results of traditional cognitive neuroscience studies. However, even if we do not adjust the original criteria, we can still draw conclusions about causation from brain stimulation studies.

## Articulating the Mind–Brain Relation

The conclusions drawn in the previous sections rely heavily on the assumption of a supervenience relation between brain states and mental states. We believe that most neuroscientists would agree with this assumption. Quoting one of the key textbooks in cognitive neuroscience programs: “Cognitive neuroscience is an academic field concerned with the scientific study of biological substrates underlying cognition, with a specific focus on the neural substrates of mental processes” [(17), p. 12]. This definition suggests that what lies at the heart of cognitive neuroscience is a dependency relation between mental states (“cognition”) and brain states (the “neural substrate”).

Supervenience is not a “deep” explanatory relation; however, it only indicates the presence of a dependence relation without telling us what it is (18). A common way to further explain this dependency is by appealing to the notion of *emergence*. Central to emergentism is the idea that supervenient properties are “novel” properties over and above the properties upon, which they supervene. In the context of the mental causation debate, emergentism can be understood as the more specific claim that mental states are the emergent properties of a complex physical system, which have their own causal power and cannot be reduced to the basic physical properties of this system.^2^ It is also possible to explain the dependence relation between mental and physical properties in terms of *reduction*. In contrast to emergentism, reductionism claims that supervenient properties are reducible to their base properties, and hence that mental properties are reducible to physical properties.

Thus far we have investigated whether interventionism allows us to make causal claims about the relation between mental states and brain states, given a notion of supervenience that in principle allows mental states to be causally efficacious *qua* mental. In other words, we have assumed a dependence relation between mental states and brain states that is non-reductive, in principle compatible with the notion of emergence, and less strong than an identity relation. However, some people might not agree with this characterization of the relation between mind and brain. What happens to the conclusions drawn in this paper when one wishes to assume a reductive relation between mental states and brain states instead? In what follows we will briefly explore this option and also consider the possibility of a causal relation.

### Type Identity and Functional Reduction

A radical reductive explanation of the supervenience relation is offered by the identity theory. This theory holds that mental states *are* brain states. The strongest version of the identity theory, the so-called “type-identity” theory, is reductionist in the sense that it states that specific types of mental states can be reduced to specific types of brain states (21). This theory would therefore claim that *M1* = *P1* and *M2* = *P2*. Now it becomes almost trivial to show that an intervention on *P1* can show a causal effect on *M*2: since we know that an intervention on *P1* will cause a change in *P2* (S3) and since *M2* is now the same as *P*2, we can conclude that *P1* causes *M2*. Thus, assuming a type-identity relation between brain states and mental states still allows us to draw conclusions about the causal effects of brain states on mental states from brain stimulation studies. Similarly, assuming a type-identity relation also makes it possible to draw conclusions about causal relations from traditional cognitive neuroscience studies: since we know an intervention on *P1* causes a change in *P*2 and *M1* = *P1*, we can conclude that *M1* causes *P*2.

The identity theory faces two important problems. First of all, it does not really provide us with an explanation of *why* mental states are identical with brain states. Take the claim that water is H_2_O. In this case, we can explain the properties of water in terms of the molecules that constitute it (two hydrogen atoms and a single oxygen atom) and the way they are interrelated. Stating that mental states are identical with brain states does not provide us with such an explanation. Second, there is the problem of multiple realizability (22, 23). If (at least some) mental states can be realized by different brain states, which seems plausible given what we know about the plasticity of the human brain, then they cannot be identical with specific brain states.

An alternative model of reduction, *functional reduction*, has been proposed by Kim (18, 24). According to this model, mental states can be reduced in the following way:

*Stage 1*. Define *M* in terms of its “causal role,” i.e., in terms of the causal task *C* it performs.
*Stage 2*. Identify the “realizers” of *M*, i.e., the actual mechanisms that perform causal task *C*.
*Stage 3*. Develop an explanatory theory that explains how the realizers of *M* perform causal task *C*.

The causal claims about brain stimulation studies and traditional cognitive neuroscience studies that can be made on the basis of functional reductionism are similar to those that can be made on the basis of the identity theory. Furthermore, functional reductionism does provide an explanation of how mental states are realized by brain states, and it is entirely consistent with the phenomenon of multiple realizability (in the sense that Stage 2 anticipates the existence of multiple lower-level realizers).

However, functional reductionism, like the identity theory, comes at a high price: it grants mental states causal power, but only in virtue of their being physical states. And this might be a hard pill to swallow, since many people believe that mental states do have causal power of their own, *qua* mental. It is precisely this intuition that is behind the debate between Baumgartner and Woodward in the first place.

### A Causal Relation

The second alternative that we will consider is one that postulates a causal relation between brain states and mental states, in the sense that *P2* causes *M2*. Although most philosophers reject this possibility [with the notable exception of (25)], it might strike cognitive neuroscientists as a plausible option.

What can we conclude from brain stimulation studies if we assume that the relation between *P2* to *M2* is a causal relation (one that has been established by means of an appropriate intervention)? In particular, can we still make causal claims about the relation between *P1* and *M2*? At first glance, the problem seems to be that *P2* is now on a directed path to *M2* that does not include *P1*. However, it is precisely this relation that allows us to conclude that *P1* is a *contributing* cause according to the second part of (M):

A necessary and sufficient condition for *X* to be a (type-level) *contributing cause* of *Y* with respect to variable set **V** is that (i) there be a directed path from *X* to *Y* such that each link in this path is a direct causal relationship… and that (ii) there be some intervention on *X* that will change *Y* when all other variables in **V** that are not on this path are held fixed [(5), pp. 59].

Note that this was not possible when we assumed a supervenience relation between *P2* and *M2* because in that case not every link on the path from *P1* to *M2* was a direct causal relation.

Unfortunately, this does not work when we want to make causal inferences from traditional cognitive neuroscience studies. *M1* cannot have a causal effect on *P2*, because it is impossible to intervene on *M1* without violating the second requirement of (IV):

2 *I* acts as a switch for all the other variables that cause *X*. That is, certain values of *I* are such that when *I* attains those values, *X* ceases to depend on the values of other variables that cause *X* and instead depends only on the value taken by *I*.

Criterion 2 is violated because of the assumption that *M1* is caused by *P1*. What this seems to show is that the assumption of a causal relation between brain states and mental states ultimately leads to *epiphenomenalism*, i.e., the thesis that mental states can be causally influenced by physical states, but have no causal efficacy themselves. It is probably safe to assume that most people will not really consider this an improvement over the minimal notion of mental causation provided by type identity and functional reduction.

## Conclusion

The aim of this paper was to investigate whether we can draw conclusions about causal relations between brain states and mental states from traditional cognitive neuroscience studies and brain stimulation studies, given an interventionist account of causation. We have argued that, if one follows Woodward in embracing the notion of supervenience and revising the criteria for what counts as an intervention, both types of studies can be used to establish causal claims. If, by contrast, one follows Baumgartner and his causal exclusion argument, traditional cognitive neuroscience studies cannot be used to establish causal claims but brain stimulation studies can.

Brain stimulation is being used more and more as a form of treatment for psychiatric disorders. We have shown that from an interventionist point of view, it is valid to say that these brain stimulation treatments *cause* changes in mental states. Now this is not necessarily an argument in favor of these treatments. However, if Baumgartner is right, then it seems reasonable to conclude that brain stimulation treatments will become increasingly attractive. Unlike traditional cognitive neuroscience studies, they actually have the potential to elucidate the causal structure of certain psychiatric disorders (i.e., the underlying causal relations between mental states and brain states). It is a safe bet that this will appeal to many psychiatrists.

At the same time, this conclusion is based on a “conservative” interpretation of interventionism, and a rejection of non-causal metaphysical relations between mental states and brain states such as supervenience. In this respect, Woodward’s position is much more “liberal,” insofar as it proposes adjusted intervention criteria and allows for the inclusion of non-causal relations between mental states and brain states. One advantage of Woodward’s position is that it allows psychiatrists to draw conclusions about the causal structure of mental disorders from traditional cognitive neuroscience (and not just brain stimulation studies). Another and perhaps even more important advantage is that it legitimates the claim that cognitive and behavioral therapy, aiming at influencing the mental state of a patient, can *cause* changes in the patient’s brain state.

A note of caution is required when applying our conclusions to psychiatric practice. When defining mental states and brain states as separate targets of intervention, we assume an ideal situation in which such a separation can be easily obtained, and Woodward’s intervention criteria are met. In practice, however, such a situation might be difficult to achieve. For example, in their paper on degeneracy, Price and Friston (26) have argued that different neural configurations can lead to similar mental states. This means that a disruption of one of these configurations by brain stimulation might not necessarily lead to a change in mental state. The experimenter who uses interventionism in the context of a single study would then be forced to conclude that there is no causal relation between the brain state and mental state in question. Now this scenario could be avoided by making sure that conclusions about causal relations between mental states and brain states are supported by multiple studies (controlling for both inter- and intra-individual variation). However, there might be a larger worry here, not just about the fact that the application of interventionism to single studies in practice might sometimes result in misguided causal claims, but also about the *very possibility* of applying interventionism to cognitive neuroscience studies.

For example, one might argue that various factors such as degeneracy, redundancy, path-dependency, non-linearity and complex feedback loops make it (theoretically) impossible to establish linear causal chains.^3^ In the light of this, several theorists have proposed a concept of “circular causation” (27–29). Circular causation, which is taken to be typical of a self-organizing system, is realized by the cooperation of the individual parts of the system, yet it also governs or constrains the behavior of these individual parts. A good illustration of circular causation is given by McGilchrist (30) in his account of the brain as a complex system: “Events anywhere in the brain are connected to, and potentially have consequences for, other regions, which may respond to, propagate, enhance or develop that initial event, or alternatively redress it in some way, inhibit it, or strive to re-establish equilibrium. There are no bits, only networks, an almost infinite array of pathways” (2010, p. 34).

**Figure 5:**
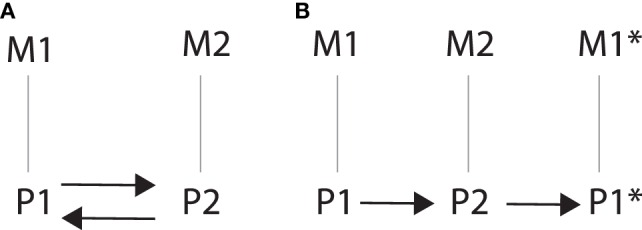
**(A)** Incorrect depiction of circular causation that fails to take into account the temporal relation between P1 and P2, and P2 and P1. **(B)** Correct depiction of circular causation that takes into account the spatial as well as the temporal relation between P1 and P2, and P2 and P1*, and illustrates the linear nature of circular causation.

Circular causation is attractive, but also slightly misleading – at least when it is articulated in opposition to linear causation. As Von Bertalanffy (31, 32) already pointed out, to make sense of circular causation we still require a notion of linear and “unidirectional” causation. The kind of feedback regulation that is implied by circular causation is obviously not unidirectional in *spatial* terms: it moves back and forth or circles around the various components of a system (33). However, despite circling in space, feedback still proceeds forward in *linear time*, one component being separated from the next in time. Circular causation, thus understood, is compatible with the assumption of a supervenience relation between mental states and brain states. The question is whether it is also compatible with interventionism. Let us say we propose a modified version of Figure 1 that involves feedback loops, for example one in which P1 causes P2 which in turns causes *P1* (Figure 5A). Now, at first glance, such a feedback loop seems to violate the second requirement of (IV), in the sense that one might think that *P1* not only depends on the value taken by *I* but also on the value of *P2*. However, the problem is that such a depiction of circular causation fails to take into account the fact that the relations between *P1* and *P2*, and *P2* and *P1* are temporal relations between different *events*, and therefore they cannot be circular. That is, the *P1* that causes *P2* is different (not spatially, but temporally) from the *P1* that is caused by *P2* as the result of the feedback loop. The correct (linear) way to represent circular causation is shown in Figure 5B: *P1* causes *P2*, and *P2* causes P1* (which is temporally different, but spatially identical with *P1*). And this seems to be compatible with interventionism, to the extent that an intervention on *P1* does not violate the second requirement of (IV).

It is important to note, at this point, that interventionism as such is relatively free of metaphysical commitments, in the sense that it does not make claims about how exactly one should spell out the relation between mental states and brain states. It only tells us what needs to be in place and which conditions need to be met for a given relation between variables to be described as “causal.” Furthermore, as we have shown in Section “The Interventionist Account of Causation,” one of the main attractions of interventionism is that it seems to correspond to how causal relations are investigated in scientific practice. Therefore, even if interventionism turns out to be incompatible with certain assumptions about brain functioning, such as circular causation and feedback loops, then this indicates a larger problem with mainstream scientific method and textbook accounts of experimental research. Obviously, these are issues that deserve critical attention. For the purpose of this paper, however, we have taken the mainstream scientific method as our starting point.

In the end, how psychiatrists approach these issues will probably depend on their intuitions about interventionist causation and the relation between mind and brain. However, one thing is certain. What they conclude will have important implications for the way they communicate the effect of different treatments to their patients.

## Author Contributions

ND wrote the core sections of this paper (“The Interventionist Account of Causation,” “Mental States and Brain States: a Supervenience Relation,” “Causation in Traditional Cognitive Neuroscience Studies,” and “Causation in Brain Stimulation Studies”) and the main argument; LB wrote the other Sections “Introduction,” “Articulating the Mind–Brain Relation,” and “Conclusion,” revised/rewrote Sections “The Interventionist Account of Causation,” “Mental States and Brain States: a Supervenience Relation,” “Causation in Traditional Cognitive Neuroscience Studies,” and “Causation in Brain Stimulation Studies and supervised the project.”

## Conflict of Interest Statement

The authors declare that the research was conducted in the absence of any commercial or financial relationships that could be construed as a potential conflict of interest.

## Footnotes

^1^Supervenience is non-causal because it represents a synchronic rather than a diachronic relation between M and P.

^2^See, e.g., Ref. (19, 20) for specific accounts of supervenient emergentism in (cognitive) neuroscience.

^3^We thank an anonymous reviewer for bringing this to our attention.
